# Numerical data concerning wind farm layout optimization using differential evolution algorithm at different wind speeds

**DOI:** 10.1016/j.dib.2017.09.040

**Published:** 2017-09-22

**Authors:** Shafiq-ur-Rehman Massan, Asim Imdad Wagan, Muhammad Mujtaba Shaikh, Muhammad Saleh Shah

**Affiliations:** a Shaheed Zulfikar Ali Bhutto Institute of Science and Technology, Karachi, Pakistan; b Mohammad Ali Jinnah University, Karachi, Pakistan; c Department of Basic Sciences and Related Studies, Mehran University of Engineering and Technology, Jamshoro, Pakistan; d Government Saifee Zahabi Edhi College of Education, Karachi, Pakistan

## Abstract

In this work, the numerical data related to wind turbine micrositing problem is presented. The data is acquired using the differential evolution algorithm (DEA) at different wind speeds. The data obtained through DEA include total dissipated power, cost per installation of unit turbine, and the efficiency of algorithm after installation of any particular number turbines; and are depicted versus number of turbines. The data provided in this paper can be used directly without having to spend weeks of computational time to simulate the results; and can readily be used for comparison with other existing (Massan et al. [1] and Rajper et al. [2], etc.) and forthcoming algorithms in future.

**Specifications Table**

**Table:** 

Subject area	*Wind Turbine Micrositing (WTM) and Wind Turbine Optimization (WTO)*
More specific subject area	*Application of Algorithms to WTM/WTO*
Type of data	*Tables and figures*
How data was acquired	*Simulation on a Core i3 Computer*
Data format	*Filtered and analyzed*
Experimental factors	Parameter values for DEA experiment for WTM,
	*a*=0.326795,
	*α*=0.09437,
	*r*_r_=40 m,
	*C*_T_=0.88,
	*X*=200 m,
	*U*_0_=12 m/s, 10 m/s, 8 m/s and 6 m/s,
	*Z*=60 m,
	*Z*_0_=0.3,
	The configuration of DEA being,
	Population size (**nP**)=100,
	Feed slave process (**feedSlaveProc**)=5,
	Maximum iterations (**maxiter)**=900,
	Maximum time (**maxtime**)=900.
Experimental features	*Computer Simulation on Matlab 2012 with DEA code*
Data source location	*Computer Simulation*
Data accessibility	*Data is gathered in appendix to this article as*Supplementary data file

**Value of the data**

• Extensive computational effort that was used to simulate the data presented in this paper saves the time of other researchers who may need to apply this data for comparison with other algorithms.
• The Differential Evolution algorithm (DEA) is one of the classical and most widely used techniques for solving the Constraint Optimization Problem of Wind Turbine Optimization, and as such the data is useful for ready comparison with upcoming algorithms.
• The results obtained herein are important as they can be directly compared with other algorithms ([1],[2]), across the board, as it is run on a standard code given by Mittal [4].
• It is suggested that other researchers may apply other algorithms to the WTO problem and compare their results with these ones as it is on a standard benchmark.
• Moreover, sufficient research avenues are available for testing new algorithms and their performance may be measured by the use of this data.

## 1 Data

The study of wind farms and the optimal placement of wind turbines to maximize the power and minimize the cost per installation of unit turbines have been explored in many works. Many algorithms have been used to solve the wind turbine micrositing (WTM) problem, and as a result the data concerning the power produced and the cost per unit turbine, versus the number of turbines by different algorithms is important; especially in the current of era of computational maturity and recent increasing trends in the new optimization algorithms.

The data describes the power produced, cost per unit turbine and efficiency of the algorithm, versus the number of turbines when WTM problem is solved using DEA at different wind speeds. Thus the effect of wind speed on the power output of the wind farm is outlined. As we know from theory, that power is a function of the cube of the wind speed, which proves true here. The power produced from the turbine is in (kW h) kilowatt-hours and the cost is a dimensionless entity without units as described by Mittal [4].

## 2 Experimental design, materials and methods

The code for the experiment is available in Mittal's [4] work and from MATLAB Central [5]. The Code of DEA was used to solve WTO problem with Jensen's model [3] and the data was generated at four different wind speeds: 6 m/s, 8 m/s, 10 m/s and 12 m/s. The complete data for the wind speeds: 6 m/s, 8 m/s and 10 m/s are shared in Tables A1–A3 in the Appendix to this data article as supplementary data file. On the hand, complete data corresponding to the speed 12 m/s was included in the study [3] and compared to genetic algorithm [2] in the same.

To note the data described in Tables A1–A3 of Appendix, following parameter values for DEA experiment were used for WTO problem [3]:

*a*=0.326795,

*α*=0.09437,

*r*_r_=40 m,

*C*_T_=0.88,

*X*=200 m,

*U*_0_=12 m/s, 10 m/s, 8 m/s and 6 m/s,

*Z*=60 m,

*Z*_0_=0.3,

The configuration of the DEA was set as follows:

Population size (**NP**)=100,

Feed slave process (**feedSlaveProc**)=5,

Maximum iterations (**maxiter**)=900,

Maximum time (**maxtime**)=900.

**Fig. 1:**
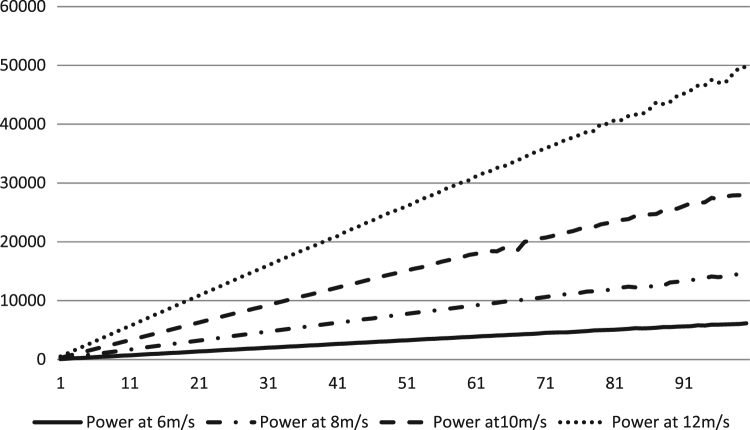
The power (kW h) produced versus number of turbines at different speeds (m/s) using DEA.

**Fig. 2:**
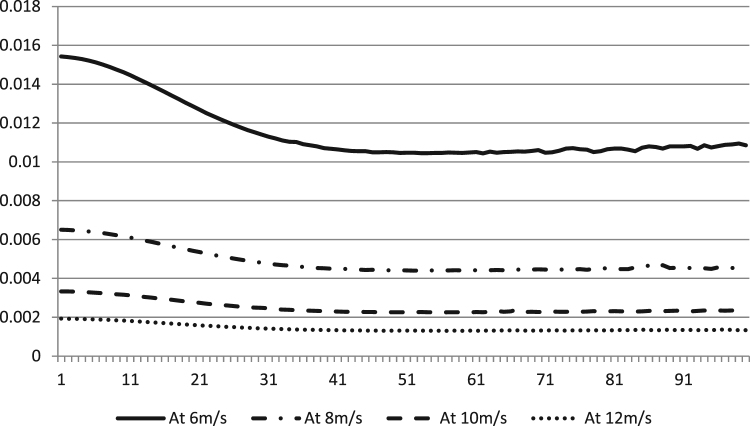
The dimensionless cost per unit turbine versus number of turbines at different speeds (m/s) using DEA.

**Fig. 3:**
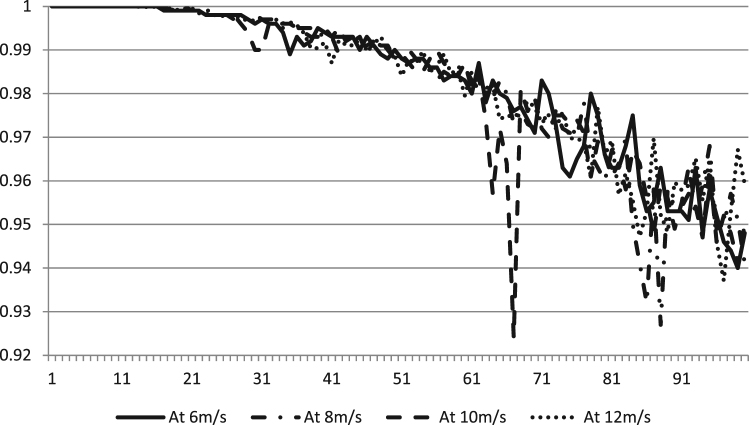
The efficiency (per unit) of the algorithm versus number of turbines at different speeds (m/s) using DEA.

After extended simulation for each wind speed and under above-mentioned specifications the data gathered in Appendix (Tables A1–A3) were acquired. Fig. 1 shows the power output data versus the number of turbines at all four wind speeds. The power increases cubically with respect to wind speed. Fig. 2 shows the dimensionless cost per unit turbine versus the number of iterations at different wind speeds. The efficiency data of the algorithm at different wind speeds are shown in Fig. 3.

Tables A1–A3 of Appendix contain the original data shared in this paper that was obtained using DEA at the mentioned wind speeds. If the power produced per number of turbines after installation of particular turbines (for example, say 33) is less than the power produced per number of turbine for installing a higher number of turbines (say 34) in Tables A1–A3 in Appendix, then this state can be interpreted as local optima. Similarly, the case with cost can be interpreted. For example, if the cost for 34 turbines say, was higher as compared to 35 turbines, assume, then again the algorithm reached local optima. Higher efficiency reported show that global optimum is arrived and the lower one means that local optimum was reached and global optimum was missed.

## Appendix A Supplementary material

Tables 1–3 containing the comprehensive data regarding power produced, cost per unit turbine and efficiency of the DEA algorithm are included in the appendix as supplementary data.
