# Supplementary material for: Numerical data concerning wind farm layout optimization using differential evolution algorithm at different wind speeds
Source: Data Brief. 2017 Sep 22;15:244–8. doi: 10.1016/j.dib.2017.09.040 (PMC5635204; doi:10.1016/j.dib.2017.09.040)
Supplement: Supplementary file 1 — Transparency document [file mmc1.docx]

**CONFLICT OF INTEREST STATEMENT**

**Title: Numerical Data concerning wind farm layout optimization using differential evolution algorithm at different wind speeds.**

We, the authors of this data article, state that we don’t have any conflict of interest with any researcher, editorial member, reviewer, organization, etc.

Regards,

Muhammad Mujtaba Shaikh (corresponding author)

Email: [mujtaba.shaikh@faculty.muet.edu.pk](mailto:mujtaba.shaikh@faculty.muet.edu.pk)

Cell # +92-333-2617602
