# Supplementary material for: Numerical data concerning wind farm layout optimization using differential evolution algorithm at different wind speeds
Source: Data Brief. 2017 Sep 22;15:244–8. doi: 10.1016/j.dib.2017.09.040 (PMC5635204; doi:10.1016/j.dib.2017.09.040)
Supplement: Supplementary file 2 — Tables 1–3 containing the comprehensive data regarding power produced, cost per unit turbine and efficiency of the DEA algorithm are included in the appendix as supplementary data. [file mmc2.docx]

**APPENDIX - SUPPLEMENTARY DATA**

**for the paper**

**Numerical Data concerning wind farm layout optimization using differential evolution algorithm at different wind speeds.**

**Table 1**

Complete results of application of Differential Evolution Algorithm to the WTO problem at a wind speed of 6 m/s

| Number of  turbines | Power produced  (kWh) | Cost per unit turbine  (dimensionless) | Efficiency  (per unit) |
| --- | --- | --- | --- |
| 1 | 64.80 | 0.0154232 | 1.000 |
| 2 | 129.60 | 0.0153964 | 1.000 |
| 3 | 194.40 | 0.0153522 | 1.000 |
| 4 | 259.20 | 0.0152909 | 1.000 |
| 5 | 324.00 | 0.0152131 | 1.000 |
| 6 | 388.80 | 0.0151198 | 1.000 |
| 7 | 453.60 | 0.0150117 | 1.000 |
| 8 | 518.40 | 0.0148900 | 1.000 |
| 9 | 583.20 | 0.0147559 | 1.000 |
| 10 | 648.00 | 0.0146106 | 1.000 |
| 11 | 712.80 | 0.0144555 | 1.000 |
| 12 | 777.60 | 0.0142920 | 1.000 |
| 13 | 842.30 | 0.0141233 | 1.000 |
| 14 | 907.10 | 0.0139472 | 1.000 |
| 15 | 972.00 | 0.0137656 | 1.000 |
| 16 | 1,036.49 | 0.0135871 | 1.000 |
| 17 | 1,100.88 | 0.0134079 | 0.999 |
| 18 | 1,165.42 | 0.0132265 | 0.999 |
| 19 | 1,230.51 | 0.0130401 | 0.999 |
| 20 | 1,294.70 | 0.0128656 | 0.999 |
| 21 | 1,359.35 | 0.0126897 | 0.999 |
| 22 | 1,424.63 | 0.0125125 | 0.999 |
| 23 | 1,488.04 | 0.0123567 | 0.998 |
| 24 | 1,552.27 | 0.0121992 | 0.998 |
| 25 | 1,616.69 | 0.0120465 | 0.998 |
| 26 | 1,681.12 | 0.0119007 | 0.998 |
| 27 | 1,745.60 | 0.0117618 | 0.998 |
| 28 | 1,810.88 | 0.0116254 | 0.998 |
| 29 | 1,872.75 | 0.0115183 | 0.997 |
| 30 | 1,936.97 | 0.0114038 | 0.996 |
| 31 | 2,002.98 | 0.0112870 | 0.997 |
| 32 | 2,065.20 | 0.0111994 | 0.996 |
| 33 | 2,130.14 | 0.0111043 | 0.996 |
| 34 | 2,191.06 | 0.0110371 | 0.994 |
| 35 | 2,243.65 | 0.0110167 | 0.989 |
| 36 | 2,316.24 | 0.0109049 | 0.993 |
| 37 | 2,377.17 | 0.0108557 | 0.991 |
| 38 | 2,441.65 | 0.0107960 | 0.992 |
| 39 | 2,515.51 | 0.0107023 | 0.995 |
| 40 | 2,576.95 | 0.0106679 | 0.994 |
| 41 | 2,637.98 | 0.0106395 | 0.993 |
| 42 | 2,703.24 | 0.0105985 | 0.993 |
| 43 | 2,768.25 | 0.0105630 | 0.993 |
| 44 | 2,829.98 | 0.0105437 | 0.993 |
| 45 | 2,886.96 | 0.0105448 | 0.990 |
| 46 | 2,958.57 | 0.0104959 | 0.993 |
| 47 | 3,019.51 | 0.0104881 | 0.991 |
| 48 | 3,074.83 | 0.0105015 | 0.989 |
| 49 | 3,137.17 | 0.0104926 | 0.988 |
| 50 | 3,206.94 | 0.0104612 | 0.990 |
| 51 | 3,265.67 | 0.0104677 | 0.988 |
| 52 | 3,326.50 | 0.0104685 | 0.987 |
| 53 | 3,393.08 | 0.0104526 | 0.988 |
| 54 | 3,458.00 | 0.0104432 | 0.988 |
| 55 | 3,513.60 | 0.0104626 | 0.986 |
| 56 | 3,576.36 | 0.0104612 | 0.986 |
| 57 | 3,631.98 | 0.0104809 | 0.983 |
| 58 | 3,696.49 | 0.0104754 | 0.984 |
| 59 | 3,763.36 | 0.0104639 | 0.984 |
| 60 | 3,821.35 | 0.0104775 | 0.983 |
| 61 | 3,873.59 | 0.0105065 | 0.980 |
| 62 | 3,964.88 | 0.0104314 | 0.987 |
| 63 | 3,991.72 | 0.0105271 | 0.978 |
| 64 | 4,075.56 | 0.0104731 | 0.983 |
| 65 | 4,126.79 | 0.0105039 | 0.980 |
| 66 | 4,185.53 | 0.0105151 | 0.979 |
| 67 | 4,238.24 | 0.0105411 | 0.976 |
| 68 | 4,306.21 | 0.0105291 | 0.977 |
| 69 | 4,354.71 | 0.0105646 | 0.974 |
| 70 | 4,402.71 | 0.0106006 | 0.971 |
| 71 | 4,521.07 | 0.0104703 | 0.983 |
| 72 | 4,573.62 | 0.0104956 | 0.980 |
| 73 | 4,602.94 | 0.0105734 | 0.973 |
| 74 | 4,616.59 | 0.0106865 | 0.963 |
| 75 | 4,671.00 | 0.0107046 | 0.961 |
| 76 | 4,754.83 | 0.0106561 | 0.965 |
| 77 | 4,828.44 | 0.0106316 | 0.968 |
| 78 | 4,951.45 | 0.0105021 | 0.980 |
| 79 | 4,991.01 | 0.0105524 | 0.975 |
| 80 | 5,006.33 | 0.0106533 | 0.966 |
| 81 | 5,056.54 | 0.0106793 | 0.963 |
| 82 | 5,114.55 | 0.0106885 | 0.963 |
| 83 | 5,207.61 | 0.0106255 | 0.968 |
| 84 | 5,307.64 | 0.0105508 | 0.975 |
| 85 | 5,283.75 | 0.0107247 | 0.959 |
| 86 | 5,309.04 | 0.0107992 | 0.953 |
| 87 | 5,386.59 | 0.0107675 | 0.955 |
| 88 | 5,490.67 | 0.0106848 | 0.963 |
| 89 | 5,494.48 | 0.0107987 | 0.953 |
| 90 | 5,559.45 | 0.0107924 | 0.953 |
| 91 | 5,618.81 | 0.0107971 | 0.953 |
| 92 | 5,671.19 | 0.0108149 | 0.951 |
| 93 | 5,806.40 | 0.0106779 | 0.963 |
| 94 | 5,775.67 | 0.0108501 | 0.948 |
| 95 | 5,896.60 | 0.0107407 | 0.958 |
| 96 | 5,923.21 | 0.0108049 | 0.952 |
| 97 | 5,944.25 | 0.0108789 | 0.946 |
| 98 | 5,995.89 | 0.0108963 | 0.944 |
| 99 | 6,029.32 | 0.0109465 | 0.940 |
| 100 | 6,145.24 | 0.0108485 | 0.948 |

**Table 2**

Complete results of application of Differential Evolution Algorithm to the WTO problem at a wind speed of 8 m/s

| Number of  turbines | Power produced  (kWh) | Cost per unit turbine  (dimensionless) | Efficiency  (per unit) |
| --- | --- | --- | --- |
| 1 | 153.60 | 0.0065066 | 1.000 |
| 2 | 307.20 | 0.0064954 | 1.000 |
| 3 | 460.80 | 0.0064767 | 1.000 |
| 4 | 614.40 | 0.0064508 | 1.000 |
| 5 | 768.00 | 0.0064180 | 1.000 |
| 6 | 921.60 | 0.0063786 | 1.000 |
| 7 | 1,075.20 | 0.0063331 | 1.000 |
| 8 | 1,228.80 | 0.0062817 | 1.000 |
| 9 | 1,382.40 | 0.0062251 | 1.000 |
| 10 | 1,536.00 | 0.0061638 | 1.000 |
| 11 | 1,689.60 | 0.0060984 | 1.000 |
| 12 | 1,843.20 | 0.0060294 | 1.000 |
| 13 | 1,996.80 | 0.0059575 | 1.000 |
| 14 | 2,150.40 | 0.0058833 | 1.000 |
| 15 | 2,303.63 | 0.0058083 | 1.000 |
| 16 | 2,456.41 | 0.0057331 | 1.000 |
| 17 | 2,610.20 | 0.0056549 | 1.000 |
| 18 | 2,762.86 | 0.0055791 | 0.999 |
| 19 | 2,916.78 | 0.0055013 | 0.999 |
| 20 | 3,070.21 | 0.0054254 | 0.999 |
| 21 | 3,222.47 | 0.0053529 | 0.999 |
| 22 | 3,374.37 | 0.0052827 | 0.999 |
| 23 | 3,529.48 | 0.0052096 | 0.999 |
| 24 | 3,681.22 | 0.0051441 | 0.999 |
| 25 | 3,833.00 | 0.0050810 | 0.998 |
| 26 | 3,985.57 | 0.0050197 | 0.998 |
| 27 | 4,134.86 | 0.0049654 | 0.997 |
| 28 | 4,291.01 | 0.0049061 | 0.998 |
| 29 | 4,441.15 | 0.0048570 | 0.997 |
| 30 | 4,596.70 | 0.0048054 | 0.998 |
| 31 | 4,746.17 | 0.0047634 | 0.997 |
| 32 | 4,900.54 | 0.0047197 | 0.997 |
| 33 | 5,051.11 | 0.0046829 | 0.997 |
| 34 | 5,196.09 | 0.0046541 | 0.995 |
| 35 | 5,350.68 | 0.0046195 | 0.995 |
| 36 | 5,501.40 | 0.0045913 | 0.995 |
| 37 | 5,652.25 | 0.0045656 | 0.995 |
| 38 | 5,809.64 | 0.0045373 | 0.995 |
| 39 | 5,950.87 | 0.0045240 | 0.993 |
| 40 | 6,106.83 | 0.0045016 | 0.994 |
| 41 | 6,255.97 | 0.0044864 | 0.993 |
| 42 | 6,382.12 | 0.0044892 | 0.989 |
| 43 | 6,553.93 | 0.0044616 | 0.992 |
| 44 | 6,703.54 | 0.0044511 | 0.992 |
| 45 | 6,863.91 | 0.0044351 | 0.993 |
| 46 | 6,984.85 | 0.0044457 | 0.989 |
| 47 | 7,158.01 | 0.0044243 | 0.992 |
| 48 | 7,305.32 | 0.0044201 | 0.991 |
| 49 | 7,455.30 | 0.0044153 | 0.991 |
| 50 | 7,585.09 | 0.0044229 | 0.988 |
| 51 | 7,749.74 | 0.0044110 | 0.989 |
| 52 | 7,906.47 | 0.0044044 | 0.990 |
| 53 | 8,042.01 | 0.0044102 | 0.988 |
| 54 | 8,185.65 | 0.0044117 | 0.987 |
| 55 | 8,329.43 | 0.0044135 | 0.986 |
| 56 | 8,455.98 | 0.0044244 | 0.983 |
| 57 | 8,625.36 | 0.0044133 | 0.985 |
| 58 | 8,764.49 | 0.0044181 | 0.984 |
| 59 | 8,937.58 | 0.0044060 | 0.986 |
| 60 | 9,089.11 | 0.0044051 | 0.986 |
| 61 | 9,206.77 | 0.0044204 | 0.983 |
| 62 | 9,337.86 | 0.0044292 | 0.981 |
| 63 | 9,499.11 | 0.0044237 | 0.982 |
| 64 | 9,619.14 | 0.0044374 | 0.979 |
| 65 | 9,813.37 | 0.0044172 | 0.983 |
| 66 | 9,937.37 | 0.0044289 | 0.980 |
| 67 | 10,017.64 | 0.0044597 | 0.973 |
| 68 | 10,197.85 | 0.0044461 | 0.976 |
| 69 | 10,315.49 | 0.0044599 | 0.973 |
| 70 | 10,450.44 | 0.0044660 | 0.972 |
| 71 | 10,614.60 | 0.0044596 | 0.973 |
| 72 | 10,799.26 | 0.0044450 | 0.976 |
| 73 | 10,930.18 | 0.0044527 | 0.975 |
| 74 | 11,086.92 | 0.0044499 | 0.975 |
| 75 | 11,214.58 | 0.0044586 | 0.973 |
| 76 | 11,319.38 | 0.0044762 | 0.970 |
| 77 | 11,562.35 | 0.0044398 | 0.978 |
| 78 | 11,569.38 | 0.0044947 | 0.966 |
| 79 | 11,687.13 | 0.0045064 | 0.963 |
| 80 | 11,795.04 | 0.0045217 | 0.960 |
| 81 | 12,036.08 | 0.0044865 | 0.967 |
| 82 | 12,205.85 | 0.0044787 | 0.969 |
| 83 | 12,357.10 | 0.0044779 | 0.969 |
| 84 | 12,254.74 | 0.0045697 | 0.950 |
| 85 | 12,286.83 | 0.0046120 | 0.941 |
| 86 | 12,307.93 | 0.0046583 | 0.932 |
| 87 | 12,804.58 | 0.0045296 | 0.958 |
| 88 | 12,530.54 | 0.0046819 | 0.927 |
| 89 | 13,093.91 | 0.0045314 | 0.958 |
| 90 | 13,194.94 | 0.0045472 | 0.954 |
| 91 | 13,337.58 | 0.0045486 | 0.954 |
| 92 | 13,518.31 | 0.0045371 | 0.957 |
| 93 | 13,612.77 | 0.0045545 | 0.953 |
| 94 | 13,849.55 | 0.0045248 | 0.959 |
| 95 | 14,117.94 | 0.0044860 | 0.968 |
| 96 | 14,000.66 | 0.0045712 | 0.949 |
| 97 | 14,153.74 | 0.0045689 | 0.950 |
| 98 | 14,405.34 | 0.0045354 | 0.957 |
| 99 | 14,465.68 | 0.0045625 | 0.951 |
| 100 | 14,461.35 | 0.0046100 | 0.941 |

**Table 3**

Complete results of application of Differential Evolution Algorithm to the WTO problem at a wind speed of 10 m/s

| Number of  turbines | Power produced  (kWh) | Cost per unit turbine  (dimensionless) | Efficiency  (per unit) |
| --- | --- | --- | --- |
| 1 | 300.00 | 0.0033314 | 1.000 |
| 2 | 600.00 | 0.0033256 | 1.000 |
| 3 | 900.00 | 0.0033161 | 1.000 |
| 4 | 1,200.00 | 0.0033028 | 1.000 |
| 5 | 1,500.00 | 0.0032860 | 1.000 |
| 6 | 1,800.00 | 0.0032659 | 1.000 |
| 7 | 2,100.00 | 0.0032425 | 1.000 |
| 8 | 2,400.00 | 0.0032162 | 1.000 |
| 9 | 2,700.00 | 0.0031873 | 1.000 |
| 10 | 3,000.00 | 0.0031559 | 1.000 |
| 11 | 3,300.00 | 0.0031224 | 1.000 |
| 12 | 3,600.00 | 0.0030871 | 1.000 |
| 13 | 3,900.00 | 0.0030503 | 1.000 |
| 14 | 4,199.47 | 0.0030126 | 1.000 |
| 15 | 4,500.00 | 0.0029734 | 1.000 |
| 16 | 4,798.25 | 0.0029350 | 1.000 |
| 17 | 5,097.79 | 0.0028955 | 1.000 |
| 18 | 5,396.91 | 0.0028562 | 0.999 |
| 19 | 5,697.38 | 0.0028164 | 1.000 |
| 20 | 5,997.55 | 0.0027773 | 1.000 |
| 21 | 6,292.99 | 0.0027411 | 0.999 |
| 22 | 6,594.50 | 0.0027031 | 0.999 |
| 23 | 6,889.63 | 0.0026688 | 0.998 |
| 24 | 7,189.01 | 0.0026341 | 0.998 |
| 25 | 7,482.91 | 0.0026027 | 0.998 |
| 26 | 7,782.54 | 0.0025707 | 0.998 |
| 27 | 8,080.94 | 0.0025407 | 0.998 |
| 28 | 8,368.16 | 0.0025158 | 0.996 |
| 29 | 8,649.98 | 0.0024937 | 0.994 |
| 30 | 8,909.19 | 0.0024793 | 0.990 |
| 31 | 9,203.08 | 0.0024565 | 0.990 |
| 32 | 9,563.25 | 0.0024185 | 0.996 |
| 33 | 9,862.11 | 0.0023984 | 0.996 |
| 34 | 10,162.68 | 0.0023796 | 0.996 |
| 35 | 10,455.67 | 0.0023640 | 0.996 |
| 36 | 10,743.51 | 0.0023510 | 0.995 |
| 37 | 11,028.54 | 0.0023399 | 0.994 |
| 38 | 11,325.08 | 0.0023276 | 0.993 |
| 39 | 11,618.05 | 0.0023172 | 0.993 |
| 40 | 11,940.87 | 0.0023022 | 0.995 |
| 41 | 12,212.19 | 0.0022983 | 0.993 |
| 42 | 12,537.54 | 0.0022852 | 0.995 |
| 43 | 12,801.95 | 0.0022841 | 0.992 |
| 44 | 13,102.15 | 0.0022774 | 0.993 |
| 45 | 13,381.94 | 0.0022749 | 0.991 |
| 46 | 13,682.56 | 0.0022695 | 0.991 |
| 47 | 13,987.90 | 0.0022640 | 0.992 |
| 48 | 14,280.10 | 0.0022612 | 0.992 |
| 49 | 14,555.61 | 0.0022615 | 0.990 |
| 50 | 14,825.78 | 0.0022628 | 0.988 |
| 51 | 15,130.42 | 0.0022593 | 0.989 |
| 52 | 15,399.26 | 0.0022614 | 0.987 |
| 53 | 15,655.06 | 0.0022655 | 0.985 |
| 54 | 15,954.07 | 0.0022635 | 0.985 |
| 55 | 16,296.55 | 0.0022558 | 0.988 |
| 56 | 16,624.53 | 0.0022505 | 0.990 |
| 57 | 16,882.31 | 0.0022548 | 0.987 |
| 58 | 17,139.68 | 0.0022592 | 0.985 |
| 59 | 17,417.30 | 0.0022609 | 0.984 |
| 60 | 17,769.27 | 0.0022532 | 0.987 |
| 61 | 17,951.86 | 0.0022671 | 0.981 |
| 62 | 18,293.22 | 0.0022609 | 0.984 |
| 63 | 18,417.20 | 0.0022816 | 0.974 |
| 64 | 18,377.25 | 0.0023226 | 0.957 |
| 65 | 18,955.19 | 0.0022868 | 0.972 |
| 66 | 19,090.62 | 0.0023054 | 0.964 |
| 67 | 18,564.31 | 0.0024065 | 0.924 |
| 68 | 20,009.50 | 0.0022660 | 0.981 |
| 69 | 20,211.78 | 0.0022762 | 0.976 |
| 70 | 20,557.54 | 0.0022703 | 0.979 |
| 71 | 20,700.06 | 0.0022868 | 0.972 |
| 72 | 20,955.28 | 0.0022907 | 0.970 |
| 73 | 21,377.37 | 0.0022767 | 0.976 |
| 74 | 21,582.87 | 0.0022858 | 0.972 |
| 75 | 21,846.32 | 0.0022888 | 0.971 |
| 76 | 22,208.04 | 0.0022815 | 0.974 |
| 77 | 22,438.10 | 0.0022878 | 0.971 |
| 78 | 22,494.25 | 0.0023117 | 0.961 |
| 79 | 22,959.59 | 0.0022939 | 0.969 |
| 80 | 23,187.50 | 0.0023001 | 0.966 |
| 81 | 23,360.71 | 0.0023116 | 0.961 |
| 82 | 23,717.08 | 0.0023050 | 0.964 |
| 83 | 23,851.02 | 0.0023200 | 0.958 |
| 84 | 24,375.63 | 0.0022974 | 0.967 |
| 85 | 24,571.88 | 0.0023062 | 0.964 |
| 86 | 24,662.23 | 0.0023247 | 0.956 |
| 87 | 24,731.45 | 0.0023452 | 0.948 |
| 88 | 25,328.14 | 0.0023163 | 0.959 |
| 89 | 25,516.61 | 0.0023253 | 0.956 |
| 90 | 25,625.46 | 0.0023414 | 0.949 |
| 91 | 26,070.18 | 0.0023271 | 0.955 |
| 92 | 26,575.90 | 0.0023079 | 0.963 |
| 93 | 26,616.85 | 0.0023294 | 0.954 |
| 94 | 26,709.19 | 0.0023463 | 0.947 |
| 95 | 27,454.56 | 0.0023068 | 0.963 |
| 96 | 27,234.42 | 0.0023500 | 0.946 |
| 97 | 27,700.00 | 0.0023345 | 0.952 |
| 98 | 27,872.34 | 0.0023440 | 0.948 |
| 99 | 27,935.37 | 0.0023626 | 0.941 |
| 100 | 28,489.47 | 0.0023400 | 0.950 |
